# A systematic review of the transiliac internal fixator (TIFI) for posterior pelvic injuries

**DOI:** 10.1051/sicotj/2021037

**Published:** 2021-07-26

**Authors:** Franz Müller, Bernd Füchtmeier

**Affiliations:** 1 Clinic for Trauma, Orthopaedics and Sports Medicine, Hospital Barmherzige Brüder Prüfeninger Str. 86 93049 Regensburg Germany

**Keywords:** Spinal instrumentation, TIFI, Posterior pelvic ring, Fractures, Outcome, Review

## Abstract

*Objective*: To summarize the literature on transiliac internal fixator (TIFI) indications and outcomes for treating posterior pelvic ring injuries. *Methods*: We searched databases for original publications in journals. Biomechanical and clinical studies using a TIFI for posterior pelvic ring injuries were considered for inclusion. The dates of publications that were included ranged from January 2000 until December 2020. *Results*: A total of 13 articles were reviewed, including eight clinical studies and five biomechanical tests. We found only case series and no multicenter or randomized study. The clinical studies contained data for a total of 186 cases, including indications, treatments, complications, and outcomes, with a minimum follow-up time of 12 months. All studies reported superior results according to operation time, blood loss, complication, dislocation, and union. One biomechanical test evaluated inferior results. *Conclusions*: The TIFI is a user-friendly and safe device to treat posterior pelvic injuries. It can also be used for acute, high-impact injuries, and fragility fractures. Nevertheless, there is no evidence concerning which types of pelvic fractures are most beneficial. Therefore, further biomechanical and clinical studies are necessary to resolve this question.

## Introduction

Unstable pelvic ring fractures are common high-impact injuries, resulting in an in-hospital mortality rate of about 8% [1]. Survivors have high morbidity and diminished quality of life [2]. Apart from spinopelvic fixation, other devices were used for the definitive treatment of posterior pelvic ring fractures.

Open reduction and internal fixation of the pelvic ring with two anterior plates crossing the iliosacral (IS) joint are common [3]. Indications for this device are IS dislocations or transalar sacral fractures according to the Arbeitsgemeinschaft Osteosynthese (AO) or Orthopaedic Trauma Association (OTA) classification [4].

Perhaps the most common procedure used to treat pelvic ring fractures is the minimally invasive stabilization of the posterior pelvis with one or two IS screws from the posterolateral side of the iliac pelvic bone into the first or second sacral body [5–7]. The third device is a bridging plate that crosses the spinous processes of the sacrum [8, 9]. Both ends of the plate are bent and fixed with several screws at the posterior iliac crest. Indications for the two devices are any type of uni- or bilateral sacral fractures, as classified by AO/OTA [4]; however, patients often require additional procedures to stabilize the anterior pelvic ring [10].

**Figure 1:**
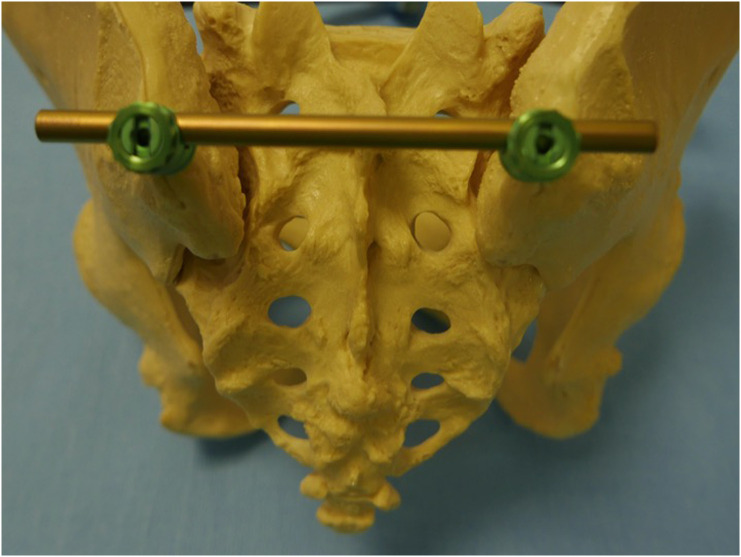
TIFI device on a bone model.

A relatively unknown technique for stabilizing the posterior pelvic ring is the placement of pedicle screws in both posterior iliac crests, combined with a transverse rod crossing the midline of the posterior sacrum (Figure 1). This minimally invasive device is often called a transiliac internal fixator (TIFI).

The primary aim of this review was to summarize the indications, techniques, postoperative care, complications, and outcomes of the TIFI for treating posterior pelvic injuries. The secondary aim was to summarize biomechanical tests using the TIFI. This is the first review for TIFI to treat posterior pelvic ring fractures.

## Material and methods

According to the MOOSE Statement guidelines, a systematic review was performed. We searched the literature for studies from January 1, 2000, through December 31, 2020. Studies covering surgeries or treatment for posterior or dorsal pelvic ring fractures using spinal instrumentations were identified from PubMed/Medline, Embase, Scopus, Google Scholar and reference lists of the selected publications. The initial search terms were as follows: pelvic/sacral fractures, posterior or dorsal stabilization/treatment, internal fixator, spinal instrumentation, TIFI, and screw-rod fixator. After excluding two case reports and two book chapters, we identified 16 studies of spinal instrumentation for posterior pelvic ring fractures, including 11 case series, and five biomechanical test series. We identified no randomized or multicenter studies.

Three case series reported previously published results (Füchtmeier et al. [11] and Dienstknecht et al. [12]; Salasek et al. [13, 14]; and Schmitz et al. [15, 16]). We excluded duplicate publications and analyzed only the recent outcomes [12, 14, 16].

In total, we analyzed eight retrospective case series (Table 1) and five biomechanical test series (Table 2).

**Table 1:** Clinical studies with TIFI and reported data for this review.

Authors	Number of cases	Mean age in years (SD) or (range)	Men/Women	Fracture type(s)	Mean follow-up in months (range)	Mean operative time / min (range)	Radiographic results	Clinical outcome	Complications
Korovessis et al. [22]	14	28.3 (6.7)	9/5	8 Denis I 3 Denis II 1 Denis III 2 not reported	Retrospectively 29 (27–39)	45 (35–65)	Matta criteria: 6 very good 5 good 4 fair 0 poor (>20 mm dislocation)	d’Aubigne: 9 good 5 satisfactory	No complications
Dienstknecht et al. [12]	67	36.7 (16-76)	38/29	46 Tile C1 11 Tile C2 10 Tile C3 17 Denis I 32 Denis II 2 Denis III 16 IS dislocation	Retrospectively 37 (36-42)	29 (max. 48)	Pelvic outcome score: 45 very good 16 good 0 fair 1 poor (>5 mm displacement)	Pelvic outcome score: 19 excellent 16 good 25 fair 2 poor	4 infection 1 screw malposition 1 fracture-dislocation 1 screw loosening
Salasek et al. [14]	32	37.9 (14–73) (3 infants)	16/16	almost 61-C1.3 2 IS dislocation no further data	prospectively not reported	not reported	Pelvic outcome score: 7 very good 5 good 12 fair 1 poor (>5 mm displacement)	Maajed score: 14 excellent 4 good 5 fair 2 poor	1 infection with implant loosening
Wang et al. [19]	29	40.8 (21–72)	15/14	5 AO 61-C1 24 AO 61-B2 4 Denis I 25 Denis II	Retrospectively 38.3 (±21.3) (12–84)	28.2 (±4.6) (20–38)	Matta criteria: 11 excellent 15 good 3 fair 0 poor (>20 mm dislocation)	Maajed score: 10 excellent 16 good 3 fair 0 poor	1 screw loosening 1 removal TIFI
Wu et al. [21]	16	36.3 (20–57)	13/10	3 Tile B2 8 Tile B3 5 Tile C1	Retrospectively 15 (13–20)	24	Matta criteria: 7 excellent 6 good 3 fair 0 poor (>20 mm dislocation)	Majeed score: 8 excellent 6 good 2 fair 0 poor	2 pain at implant side
Schmitz et al. [16]	11 (25)	n.r.	n.r.	3 high impact 8 fragility fracture	Retrospectively n.r.	45 (±19)	Not reported for TIFI	Not reported for TIFI	No infection
Omar et al. [20]	11 (38)	n.r.	n.r.	not reported to TIFI	Retrospectively ≥ 12 months	n.r.	Not reported for TIFI	Not reported for TIFI	1 fracture-dislocation
Korovessis et al. [23]	6 (22)	41.6(16-67)	4/2	3 AO 61-C1 2 AO 61-C2 1 AO 61-C3	Retrospectively Not reported To single or double TIFI	76 (68–80)	Matta criteria: 5 excellent 0 good 1 fair 0 poor (>20 mm dislocation)	Maajed score: 9 excellent 14 good 4 fair 0 poor	2 infection 1 screw loosening

n.r. = not reported; Schmitz *et al*. [16], Omar *et al*. [20], and Korovessis *et al.* [23] evaluated the outcomes of different devices. Therefore, the number of patients given in brackets is the total sample size.

**Table 2:** Biomechanical test series with TIFI.

Authors	Test models	Type of sacral fracture	Vertical load	Summary/Conclusion
Dienstknecht et al. [29]	6 freshly frozen human pelvis	6 unilateral transforaminal	3 cycles of 70% of the former body mass were performed max load not reported	TIFI compared to two IS screws were tested TIFI was not inferior compared to IS screws
Salasek et al. [30]	Computed image finite element	Unilateral transforaminal	250–500 N	TIFI has enough stiffness for unstable transforaminal fractures TIFI is superior compared to two IS screws
Shinohara et al. [31]	8 synthetic pelvic models	8 unilateral transforaminal	Max. load 1057 N Mean load for a 5 mm gap: 438 N	Double (dual) TIFI with crosslinks were used TIFI was superior compared to plate
Vigdorchik et al. [32]	6 synthetic pelvic models	3 unilateral transforaminal 3 IS joint dislocation	5 cycles; static load 10 mm/sec Mean load for a 7 mm gap: 81 N (sacral fracture) Mean load for a 7 mm gap: 71 N (IS dislocation)	Sacral fracture: TIFI was inferior compared to TIFI plus IS screw Sacral fracture: TIFI was equal to IS screw alone IS dislocation: TIFI was the weakest construct
Chaiyamongkol et al. [33]	12 synthetic pelvic models	12 unilateral transforaminal	280, 161, and 510 N Static load 10 mm/min Mean load for a 5 mm gap: 287 N	TIFI without IS screw was not tested TIFI plus one IS screw better than two IS screws or plate

## Results

### Clinical studies

#### Indications

There were many indications (Table 1), including all types of unilateral sacral fractures, with alar (*n* = 29), transforaminal (*n* = 60), and central sacral fractures (*n* = 3), according to the Denis classification [17]. TIFI has been used in type B2 (*n* = 26), B3 (*n* = 8), C1 (*n* = 51), C2 (*n* = 11), and C3 fractures (*n* = 10), according to the Tile classification [18]. Regarding the AO/OTA classification [4], types B2 (*n* = 24), C1 (*n* = 8), C2 (*n* = 2), and C3 (*n* = 1) fractures were treated. Salasek et al. reported almost on treatment of type C1.3 fractures (*n* = 32) [14]. Only one study reported indications for fragility fractures (*n* = 63) [16].

#### Techniques

**Figure 2:**
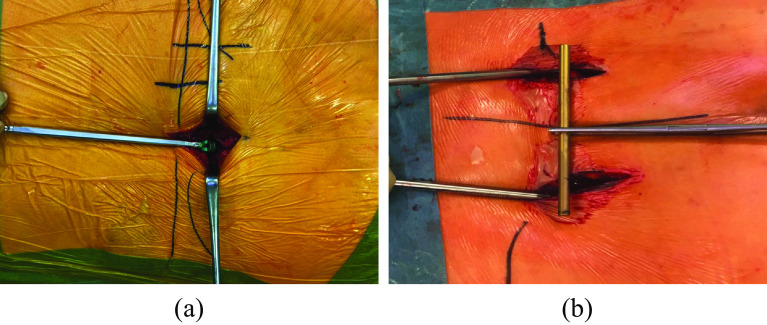
(a) In situ placement of the first pedicle screw (Univeral Spine System II; Depuy/Synthes, 79224 Umkirch, Germany). (b) TIFI after placement of both pedicle screws and before introducing the crossbar subfascial.

The techniques were different in the selected articles. Two vertical incisions for the placement of the pedicle screws were made 1 or 2 cm lateral to the posterior superior iliac spine (PSIS; Figure 2a). The incisions reported by Wang et al. were just medial to the PSIS [19], and Omar et al. used small incisions directly on the PSIS [20]. The lengths of both incisions were about 3–4 cm [12, 21], depending on proximity to the lateral border of the sacrum. The fascia was opened to the iliac pelvis to determine the entry point for the pedicle screws, located on the medial side of the dorsal iliac crest, about 1–2 cm cranial to the PSIS [21]. Reducing implant prominence was achieved by resecting some of the cortex with rongeurs or chisels, thereby placing the head of the screws deeper into the bone [21, 22].

Once the entry point was selected, the cortex was perforated with a bone awl. Next, a pedicle finder was used to create a tunnel in between both iliac cortices. Care was taken to avoid penetration of the opposite cortex at the anterior inferior iliac spine (AIIS). The same procedure was performed on the contralateral side. According to some authors [14, 23], the screw position was approximately 30° in the sagittal plane and 40° laterally, according to the pelvic configuration. All studies did not report screw direction.

In 2015, Schmitz et al. evaluated patients treated for fragility fractures [16]. Patients received a cement-augmented TIFI construction with cannulated Schanz screws. In all cases, the screw direction was from the PSIS to the AIIS of the supra-acetabular region, and the mean screw length was 100 mm (range 70–135 mm). The entry point for the screws was determined using K-wires and an intraoperative fluoroscope on an obturator oblique-outlet view, which allowed surgeons to visualize a safe corridor within the bone canal [16]. To date, cannulated screws in TIFI devices have not been reported.

Most of the authors used different solid mono- or polyaxial pedicle screws with 50–80 mm in length; and 7 mm or 7.5 mm in diameter [12, 19, 21]. After the pedicle screws were introduced, a 5.5 mm or 6 mm crossbar was inserted subfascial from one pedicle screw to the other (Figure 2b). Omar et al. introduced this crossbar only subcutaneously [20]. Once the exact length of the rod had been determined, the sleeves at the pedicle screws were connected and tightened. An assistant held the crossbar with a clamp holder to prevent shear forces while tightening the nuts. Fascia and skin closure was performed without drainage [12]. Of note, Korovessis et al. used a dual TIFI construction (four pedicle screws and two crossbars next to, but not connected) for type C3 fractures [22, 23]. Finally, the reported operation times were about 30 min, and the corresponding mean blood loss less than 100 mL [12, 16, 19, 21].

#### Complications

The complication rate between 12 to 84 months after surgery was 7.5% (*n* = 14) for a total of 186 patients in eight studies (Table 1). Infection was the most reported complication (*n* = 9) and one study reported pain at the implant site [21]. Neurovascular injuries caused by a malperforated screw or non-union were not reported.

#### Outcomes

According to the radiological outcome and the Matta criterion [24], the results were satisfactory (Table 1). Clinical evaluations were reported in six studies by three different measures. Three studies [14, 19, 23] reported the outcome with the Majeed Score [25]. The results were recorded as excellent (*n* = 32), good (*n* = 26), fair (*n* = 10), or poor (*n* = 2). Korovessis et al. used the same score and reported no poor outcomes [23]. The study of Dienstknecht et al. [12] reported excellent (*n* = 19), good (*n* = 16), fair (*n* = 25), or poor (*n* = 2) results according to the Hannover Pelvic Outcome Score [26]. Korovessis et al. [22] reported satisfied (*n* = 5) or good (*n* = 5) results according to the d’Aubergine score [27]. Two studies did not report clinical outcomes [16, 20].

#### Postoperative care

The TIFI device allowed immediate full weight-bearing on the non-injured and partial weight-bearing on the injured side [16]. Crutches or a walker should be used for about 6–8 weeks postoperatively. One surgeon prohibited any activities other than sitting until six weeks after surgery, with partial weight-bearing, resumed at six weeks, followed by complete weight-bearing activities after three months [21]. In bilateral pelvic injuries, a six-week relief period was recommended; therefore, mobilization could only be performed with a wheelchair. Prophylaxis against heterotopic bone ossifications was not recommended [19]. Elective implant removal of the TIFI could be performed 4–12 months postoperatively but could be performed later, as a part of a single session to remove more implants [12, 21].

### Biomechanical test

The test series and the used models were not standardized, and therefore, must be interpreted with caution. The first test series was presented at an international congress by Füchtmeier et al. in 2002 [28]. The abstract of this oral presentation was published in the German language [28]. Subsequently, Dienstknecht et al. published the results of this test series in detail [29]. According to AO/OTA, the authors used fresh-frozen human pelvis with a simulated type C1.2 injury [4]. The symphysis was stabilized with a plate. Three posterior devices (TIFI, two IS screws, and an anterior double plating) were tested and no significant differences in pelvic deformation were found.

Salasek et al. used a computed finite model to compare the stability of the TIFI to two IS screws in a complete unilateral transforaminal sacral fracture [30]. Compared to the IS screws, the TIFI had significantly higher stiffness, lower stress, and lower risk of over-compression. Shinohara et al. evaluated the stability of a dual TIFI construction compared to a posterior plate in synthetic pelvic models with a unilateral transforaminal sacral fracture [31]. The authors concluded that the modified dual TIFI device was more stable than the posterior plate. In 2015, Vigdorchik et al. compared TIFI, TIFI plus one IS screw, a single IS screw, and two IS screws at S1 and S2 [32]. Composite pelvis with unilateral sacral fractures and unilateral SI joint disruptions were used. In the sacral fracture model, the TIFI with an additional IS screw was the strongest device. There was no difference between a TIFI and a single S1 screw. In the IS joint disruption model, the single TIFI without any additional screw was the weakest device. Therefore, the authors did not recommend TIFI for the treatment of IS dislocation. The most recent test series was conducted by Chaiyamongkol et al. [33]. The 12 synthetic pelvic models had unilateral 5 mm gaps at the left transforaminal zone, and the pubic symphyses were separated and stabilized with plates. The TIFI with one IS screw was tested against two IS screws or a tension band plate. The TIFI was considered the best option for the treatment of vertically unstable sacral fractures.

The five biomechanical tests are summarized in Table 2.

## Discussion

### Summary

We reviewed the literature on a bilateral transiliac spinal instrument (called TIFI) to stabilize posterior pelvic ring injuries. The device can be implemented quickly and easily, is minimally invasive, and is associated with low blood loss, low complications rates, and high union rates.

### Limitations

The review of the literature related to TIFI is limited in several ways. The number of the evaluated patients included in the studies was low; therefore, statistical analysis could not be performed. To date, no randomized or multicenter study has been conducted. Furthermore, one study summarized different used devices in the analysis but did not separate their results accurately [23]. Therefore, all results must be interpreted with caution.

### Indications

**Figure 3:**
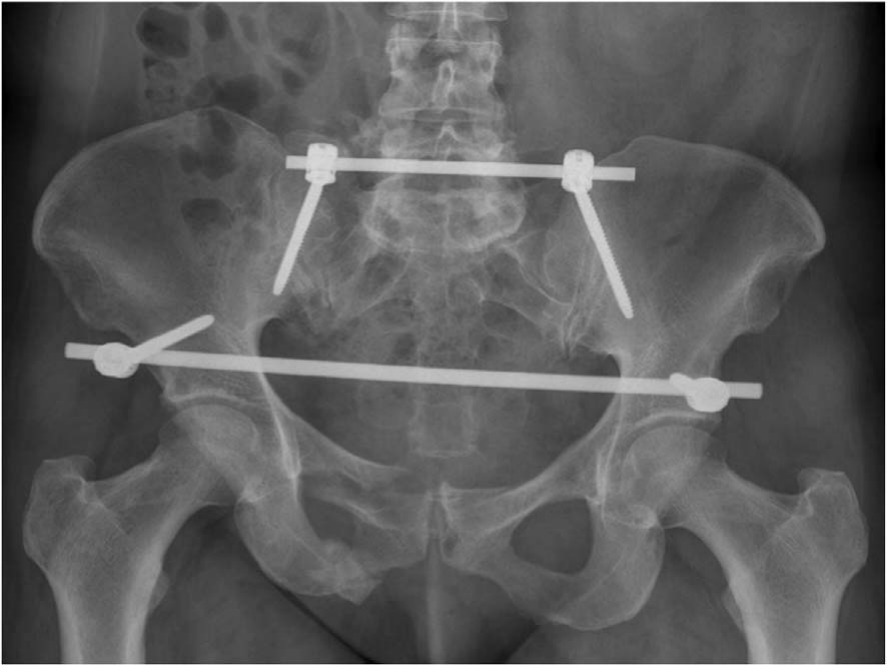
Postoperative plain radiograph demonstrated complete pelvic ring fracture at the right side with a sacral fracture treated with a TIFI and an anterior internal fixator.

From the authors’ point of view, TIFI can also be used for open fractures or after open reduction of sacral fractures; however, case series have not been reported. For severe vertical fracture dislocations with high instability, the TIFI can be extended, via rod connectors, to a uni- or bilateral iliolumbar fixator or a lumbopelvic fixator [23]. Moreover, the TIFI extension is suited for spinopelvic dissociations with different fracture patterns [23]. Additional fixation of the pelvis with an IS screw connected to a triangular fixator or additional plating of a caudal sacral fracture, is also possible. Detailed analyses of these extensions or combinations are lacking. There are no published clinical or biomechanical test series comparing the TIFI with a triangular fixation for uni- or bilateral type C3 fractures or IS dislocations. Finally, it should be noted that most anterior pelvic injuries, such as symphysis ruptures, must be treated with additional devices (e.g., external or internal fixators; Figure 3). In our opinion, relative contraindications are fractures on the dorsal part of the iliac crest and skin injuries at the incision areas, such as Morel–Lavallée lesion; however, contraindications have not been reported to date. Finally, for the placement of the device, a prone position is mandatory. With very few exceptions, such as open chest trauma, a prone position for about 30–60 min is usually not contraindicated.

### Techniques

There are several reported variations in screw construction (mono- or polyaxial screws, over-the-top loading, or side-loading), length, diameter, and placement direction. The various screws all appear to be strong enough to stabilize the posterior pelvic ring until union. According to computed tomography (CT) scan study, the anterior and posterior iliac pelvis showed an average bone canal length from the PSIS to the AIIS of 140 mm in men and 130 mm in women [34]. Minimum bone canal lengths were 120 mm for men and 90 mm for women [34]. The width of the bone canal allowed the placement of screws with a diameter of 8 mm in men and 6–7 mm in women [34]; these measures should be kept in mind.

Interestingly, one author introduced the crossbar subcutaneously [20]. However, the authors of this review stress that it is necessary to place the crossbar subfascial to reduce wound complications. In severe dislocations of the posterior pelvic ring, the reduction can be supported by manipulating the leg or placement of a Schanz screw in the pelvic wing using the “joystick technique.” We do not recommend a reduction maneuver directly with the pedicle screws because this could loosen the pedicle screws. Fracture reposition and the position of the pedicle screws can be checked using a fluoroscope; however, it is not used routinely by the authors of this review.

### Complications

The reported total complication rate of the TIFI is low (7.5%). Perhaps this is because of the simple technique with a fast operation time and reduced blood loss. The most-reported complication was infection; however, the reasons were not reported. In our opinion, an infection can be the result of the heads of the pedicle screws not being placed sufficiently deep at the iliac bone. We highly recommend placing the crossbar subfascial and not subcutaneously. Despite the learning curve being quite fast, the device is a spinal instrumentation, and therefore, it should be trained, e.g., with a pelvic bone model, before starting in situ to avoid any complications, e.g., neurovascular injuries which were not reported in any of the studies.

### Outcomes

The outcomes revealed radiologically and clinically satisfactory results. Nevertheless, it should be kept in mind that the outcome is the result of the treatment or the device and (more often) the impact of the accident, e.g., visceral or neurovascular injuries. Debate is ongoing as to how pelvic malreduction affects clinical outcomes [35]. For example, there remains no evidence for the value of performing open reduction and internal fixation of a dislocated sacral fracture or stabilizing this type of fracture pattern without reduction [36]. In our review, three clinical outcome measures were used in only six studies, suggesting that no single measure can be considered the best tool for evaluating pelvic ring injuries after surgery. Interestingly, one study used only an outcome scoring for hip arthroplasty [22].

### Biomechanical tests

The test series and the used models are not standardized, and therefore, must be interpreted with caution. Only one biomechanical test series was conducted with the human cadaver pelvis and intact ligamentous structures. Therefore, the corresponding results are difficult to compare. The other tests were performed using synthetic models or with computed finite element analysis. Various cycle loads were used (Table 2), and no tests have been performed for bilateral transforaminal sacral fractures. The biomechanical test series also demonstrated a lack of evidence to support the hypothesis that TIFI is superior to other posterior devices. Nevertheless, TIFI was inferior to other devices in only one test series. In summary, the used biomechanical loading modes do not represent the forces on the (hemi) pelvis, and therefore, the results cannot be applied to the treatment of patients. More investigations are required using similar specimens, loading arrangements, and motion measurements.

## Conclusions

TIFI is a spinal instrumentation used for the fixation of posterior pelvic injuries. It is a simple and safe technique with a short operation time; therefore, it can be used for immediate definitive interventions. There are few associated complications. Despite these advantages, it remains to determine which types of pelvic fractures require additional, stronger posterior fixations. Therefore, further biomechanical studies and clinical studies with more significant numbers of patients are necessary to provide definitive answers.

## Competing interests

The authors declare that they have no conflict of interest.

## Funding

The review did not receive any specific grant from funding agencies in the public, commercial or no-profit sectors.

## Ethical approval

The review does not contain any studies with humans or animals performed by any of the authors.

## Consent to participate

For this literature review a consent to participate was not necessary.

## Consent to publish

Consent to publish is not applicable for this study.

## Authors contributions

We thank Hannah Mueller for taking photo of the pelvis model.

## Availability of data and material

Availability of data and material is not applicable for this study.
